# Plasma IgG N-Glycosylation as a Novel Biomarker of Disease Severity and Prognosis in Diffuse Large B-Cell Lymphoma, Not Otherwise Specified

**DOI:** 10.3390/biomedicines14102150

**Published:** 2026-09-23

**Authors:** Vibor Milunović, Maja Pučić-Baković, Frano Vučković, Ivana Duvnjak-Orešković, Karla Mišura Jakobac, Marin Kursar, Inga Mandac Smoljanović, Delfa Radić-Krišto, Slavko Gašparov

**Affiliations:** 1 Division of Hematology, Clinical Hospital Merkur, 10000 Zagreb, Croatia; karlamisura@gmail.com (K.M.J.); makursar@gmail.com (M.K.); imandac@yahoo.com (I.M.S.); delfa.radic.kristo@gmail.com (D.R.-K.); 2 Glycoscience Research Laboratory, Genos Ltd., 10000 Zagreb, Croatia; mpucicbakovic@genos.hr (M.P.-B.); fvuckovic@genos.hr (F.V.); iduvnjak@genos.hr (I.D.-O.); 3 School of Medicine in Zagreb, University of Zagreb, 10000 Zagreb, Croatia; gasparovslavko@gmail.com; 4 Clinical Department of Pathology and Cytology, Clinical Hospital Merkur, 10000 Zagreb, Croatia

**Keywords:** DLBCL, IgG glycosylation, prognostic indices, biomarker, prognosis

## Abstract

**Background**: Diffuse large B-cell lymphoma (DLBCL) is a biologically heterogeneous lymphoma with variable clinical outcomes despite established molecular and clinical prognostic models. Immunoglobulin G (IgG) N-glycosylation regulates antibody effector functions and reflects systemic immune status, but plasma IgG N-glycosylation has not previously been systematically investigated in DLBCL. We evaluated whether plasma IgG N-glycosylation is altered in DLBCL and examined its associations with molecular subtype, established prognostic indices, and clinical outcomes. **Methods**: Plasma samples were obtained from 54 treatment-naïve patients with pathologically confirmed DLBCL, not otherwise specified (NOS), and 54 age- and sex-matched healthy controls. IgG N-glycans were analyzed using capillary gel electrophoresis with laser-induced fluorescence (CGE-LIF), resolving 27 glycan peaks that were summarized into six derived glycosylation traits: agalactosylation (G0), monogalactosylation (G1), digalactosylation (G2), sialylation (S), bisecting N-acetylglucosamine (B), and core fucosylation (CF). Associations with disease status, cell-of-origin subtype, prognostic indices (International Prognostic Index (IPI), Age-Adjusted International Prognostic Index (aaIPI), revised International Prognostic Index (R-IPI), National Comprehensive Cancer Network International Prognostic index (NCCN-IPI), Central Nervous System International Prognostic Index (CNS-IPI) and inflammation-based cumulative prognostic score system (ICPS)) progression-free survival (PFS), overall survival (OS), and relapse were assessed using multivariable regression and Cox proportional hazards models adjusted for age and sex. Multiple testing was controlled using the Benjamini–Hochberg false discovery rate. **Results**: Compared with healthy controls, patients with DLBCL, NOS exhibited significantly increased agalactosylation and decreased digalactosylation and sialylation, consistent with a pro-inflammatory IgG glycosylation profile. The non-germinal center B-cell-like (non-GCB) subtype showed significantly lower monogalactosylation than germinal center B-cell like (GCB) DLBCL. Across multiple established prognostic indices, increasing clinical risk was consistently associated with increased agalactosylation and reduced digalactosylation. During follow-up, 20 patients (37%) relapsed, and 21 (39%) died. Increased agalactosylation was independently associated with shorter PFS, whereas reduced sialylation predicted both inferior PFS and an increased risk of relapse. **Conclusions**: Plasma IgG N-glycosylation is profoundly altered in DLBCL, NOS and is associated with molecular subtype, established prognostic indices, and clinically relevant outcomes. These findings identify plasma IgG N-glycosylation as a promising minimally invasive biomarker that reflects disease biology and provides prognostic information beyond conventional clinical risk models. Given the exploratory nature and modest size of this cohort, these findings should be considered hypothesis-generating and require validation in larger prospective cohorts.

## 1. Introduction

Diffuse large B-cell lymphoma (DLBCL) is an aggressive lymphoid malignancy characterized by the proliferation of large neoplastic B cells and a diffuse growth pattern. It represents the most common form of non-Hodgkin lymphoma, with a worldwide incidence ranging from 2.3 to 13.8 cases per 100,000 persons annually [1]. The epidemiology of DLBCL varies substantially by age, gender, and ethnicity; the median age at diagnosis ranges from 66 to 70 years, with a slight male predominance and the highest incidence observed among non-Hispanic White populations [2]. Driven by global demographic shifts and population aging, both the prevalence and incidence of DLBCL are projected to increase in the coming years [3]. Clinically, the disease typically presents with rapidly enlarging tumors in lymph nodes, although up to 40% of patients exhibit extranodal involvement, most commonly affecting the gastrointestinal tract, skin, bone, central nervous system, or testes [4]. This aggressive clinical behavior is frequently accompanied by “B-symptoms”, including fever, night sweats, and significant unintentional weight loss, which reflect both high tumor burden and a systemic inflammation [5]. Consistent with this, inflammatory biomarkers such as C-reactive protein (CRP) and imaging-derived measures including metabolic tumor volume (MTV) have been associated with adverse clinical outcomes [6,7].

The treatment landscape of DLBCL, NOS has evolved substantially over the past decades, from CHOP (cyclophosphamide, doxorubicin, vincristine, and prednisone) chemotherapy to the incorporation of the anti-CD20 monoclonal antibody rituximab, which established R-CHOP (rituximab, cyclophosphamide, doxorubicin, vincristine and prednison) as the standard first-line treatment and markedly improved survival [4,8]. More recently, advances in molecular profiling and the development of targeted agents, such as the antibody-drug conjugate polatuzumab vedotin, which targets the B-cell antigen receptor complex-associated protein beta chain (CD79b) subunit of the B-cell receptor (BCR) ubiquitously expressed on malignant B cells, have further refined therapeutic strategies [9]. As a result, the current overall 5-year relative survival rate approaches 64.8% [10], although outcomes remain heterogenous and are strongly influenced by patient age, molecular subtype, clinical stage, and treatment intensity [4,11].

The majority of cases are classified as diffuse large B-cell lymphoma, not otherwise specified (DLBCL, NOS), a category encompassing aggressive large B-cell lymphomas that do not meet the criteria for more specific entities. DLBCL, NOS is characterized by pronounced biological and clinical heterogeneity and is further stratified based on its cell-of-origin (COO) into the germinal center B-cell-like (GCB) and activated B-cell-like (ABC) subtypes [12,13]. Although gene expression profiling remains the gold standard for COO stratification, technical requirements often necessitate the use of immunohistochemical surrogates in routine clinical practice [14]. The Hans algorithm, based on Cluster of differentiation 10 (CD10), B-cell lymphoma 6 (BCL6), and Multiple Myeloma Oncogene 1 (MUM1) expression, is the most widely used alternative method classifying cases into GCB and non-GCB (predominantly ABC) subtypes [15]. These subtypes differ substantially in their underlying biology, including BCR signaling, Nuclear factor kappa-light-chain-enhancer of activated B cells (NF-κB) activation, genetic alterations, therapeutic response, and prognosis, with the ABC subtype generally associated with inferior clinical outcomes in the rituximab era [4,16].

Despite the availability of molecular markers, clinicians continue to rely on clinical prognostic indices to evaluate risk and, in some cases, guide therapeutic decisions [17,18]. The most established tool is (IPI), developed in the early 1990s [11]. The IPI remains a cornerstone of risk stratification in the immunochemotherapy era and can be age-adjusted (aaIPI) to better suit specific patient cohorts [19]. Several refinements have been proposed to enhance its predictive power, including t R-IPI [20], NCCN-IPI [21], CNS-IPI [22], as well as (CPS [23]. However, current prognostic models rely largely on clinical parameters and fail to fully capture the biological and immunological complexity of DLBCL, NOS, highlighting the need for complementary biomarkers that more directly reflect disease biology.

Beyond tumor-intrinsic molecular features and clinical scores, increasing evidence suggests that host immune features contribute significantly to disease biology and treatment response in DLBCL, NOS [24,25]. An important yet relatively underexplored component of this host-immune interaction is protein glycosylation, the most ubiquitous and highly complex post-translational modification where carbohydrate chains (glycans) are enzymatically attached to proteins. Immunoglobulin G (IgG), the most abundant circulating antibody, is of particular clinical relevance due to its central role in humoral immunity. IgG exerts its effector functions through antigen binding and interaction with fragment crystallizable gamma receptors (FcR) and complement components. These interactions are tightly regulated by N-linked glycosylation of the IgG fragment crystallizable (Fc) region, which profoundly influences both the antibody’s structural stability and its immune effector activity [26].

Each IgG molecule contains a conserved *N*-glycosylation site at the asparagine 297 (Asn297) residue of the Fc region, while a smaller portion (15–25%) of molecules also carry additional N-glycosylation sites in the variable fragment antigen-binding region (Fab) [27]. Altered IgG glycosylation patterns have been consistently reported in immune-mediated inflammatory diseases, cardiometabolic conditions, infectious diseases, and malignancies [28]. Research has shown that IgG glycosylation is regulated by a large and complex network of genes, several of which have also been implicated with autoimmune diseases and/or hematological cancers [29,30]. In hematologic malignancies, emerging studies suggest that aberrant IgG glycosylation patterns are associated with disease activity, treatment response, and prognosis [31,32,33,34,35]. In DLBCL specifically, recent research has shown that approximately 40–50% of GCB-DLBCL cases, predominantly those belonging to the EZB genetic subtype, acquire novel *N*-glycosylation sites within the variable region of their BCR [36,37]. These sites are primarily occupied by oligomannose glycans, which are rare in normal B cells and are associated with more rapid disease progression, comparable to that observed in the high-risk ABC subtype [36,38]. Despite growing evidence implicating glycosylation in DLBCL biology, existing studies have primarily focused on tumor-cell or BCR glycosylation. In contrast, plasma IgG N-glycosylation, which may provide a systemic readout of humoral immune status, has not previously been systematically investigated in DLBCL. Given the pronounced biological heterogeneity of the disease and the central role of immune-mediated mechanisms in its pathogenesis and treatment response, comprehensive characterization of the IgG N-glycome may provide novel insights into disease biology and identify clinically relevant biomarkers.

In this study, we comprehensively characterized plasma IgG N-glycosylation in patients with DLBCL, NOS, and investigated its associations with molecular subtypes, established clinical prognostic indices, and survival outcomes. We hypothesized that plasma IgG N-glycome reflects systemic immune dysregulation accompanying DLBCL and may serve as a minimally invasive biomarker of disease severity and prognosis.

## 2. Materials and Methods

### 2.1. Subjects

This study prospectively included 54 consecutive patients with pathohistologically confirmed DLBCL, NOS and 54 age- and sex-matched healthy control subjects. The diagnosis of DLBCL, NOS was established according to the 2016 World Health Organization (WHO) classification of lymphoid neoplasms [5]. The main inclusion criterion was the ability to tolerate anthracycline-based immunochemotherapy. Exclusion criteria included: active malignant disease or a malignancy within the previous 5 years (except basal cell or squamous cell carcinoma of the skin, or cervical carcinoma in situ), active hepatitis B or C, HIV infection, active infection at diagnosis, infiltration of the central nervous system (CNS) by lymphoma, and Ann Arbor stage I/II disease with modified IPI = 1–2 without mesenteric lymphadenopathy for whom a positron emission tomography-computed tomography (PET-CT) guided approach was planned [39].

After initial laboratory (Supplementary Table S1) and ultrasound evaluation, all patients underwent multislice computer tomography (MSCT) scanning of the thorax, abdomen, and pelvis, along with bone marrow assessment, for staging according to Ann Arbor classification [40]. Patients with high suspicion of CNS involvement additionally underwent cerebrospinal fluid analysis and targeted neuroimaging.

Pathohistological diagnosis was established on hematoxylin-eosin-stained tissue sections and evaluated according to the phenotypic criteria of the 2016 World Health Organization (WHO) classification [5]. Immunohistochemical analysis was performed using antibodies against CD20, CD3, CD10, BCL6, B-cell lymphoma 2 (BCL2), MUM1, marker of proliferation Ki67 (Ki-67) and C-MYC Proto-Oncogene (c-MYC). Deparaffinization and heat-induced epitope retrieval were performed in a PT Link system (Dako, D, Agilent Technologies, Santa Clara, CA, USA) using high-pH buffer (pH 9.0) at 97 °C for 20 min. Antigen detection was carried out using a polymer-based indirect method (EnVision™ FLEX High, Dako, Agilent Technologies, Santa Clara, CA, USA), with 3,3′-diaminobenzidine (DAB) applied on a Dako Autostainer (Dako, Agilent Technologies Santa Clara, CA, USA). The GCB and non-GCB subtypes were determined according to Hans’ algorithm [15]. The Hans algorithm was used because it can be readily applied to routinely processed diagnostic tissue using standard immunohistochemistry and was consistently available across the study cohort, whereas GEP-based COO classification was not available at the participating centers during the study period. In patients with a “double-expressor” phenotype, fluorescence in situ hybridization (FISH) for MYC, BCL2, and BCL6 rearrangements was performed to exclude high-grade B-cell lymphoma.

Treatment regimens were selected by a lymphoma hematologist and included rituximab, cyclophosphamide, doxorubicin vincristine, prednisone 21 (R-CHOP-21), rituximab, dose-adjusted etoposide, prednisone, vincristine, cyclophosphamide, and doxorubicin (R-DA-EPOCH), R-mini-CHOP-21, or polatuzumab vedotin-rituximab-cyclophosphamide, doxorubicin prednisone (Pola-R-CHP). The use of biosimilar agents was allowed [41]. MSCT reevaluation was performed between the third and fourth treatment cycles. Patients with stable or progressive disease received salvage chemotherapy with the intent of undergoing autologous stem cell transplantation. Patients at high risk for CNS relapse were given two cycles of high-dose methotrexate. Radiotherapy was administered selectively according to the guidelines of the Croatian Cooperative Group for Hematologic Diseases, including patients with partial remission when feasible, those with bulky disease (≥7.5 cm), or patients with testicular lymphoma to prevent contralateral testicular involvement [42].

Treatment response was assessed using the 5-point Deauville score in accordance with the Lugano classification [43]. Patients not achieving complete metabolic remission and whose disease was not amenable to consolidative radiotherapy received platinum-based salvage therapy with intent for autologous stem cell transplantation.

Patients and control subjects were recruited at Clinical Hospital Merkur. Blood samples from patients were collected at a single time point prior to therapy initiation, and no corticosteroid prephase was permitted. Control samples were obtained during evaluation for suspected coronary artery disease, which was subsequently ruled out. All subjects provided written informed consent. The study was approved by the University of Zagreb, School of Medicine, Zagreb, Croatia (code number 641-04/23-02/) and conducted in accordance with the Declaration of Helsinki.

### 2.2. Plasma Isolation

All subjects had a peripheral venous blood sample collected in vacuum blood collection ethylenediaminetetraacetic acid (EDTA) tubes. The blood was allowed to rest for an hour at room temperature and was then centrifuged at 1620× *g* for 10 min. Plasma was then transferred to a 2 mL tube and centrifuged at 2700 *g* for 10 min and after that immediately stored at −20 °C until further processing.

### 2.3. IgG Isolation

IgG was isolated from plasma by affinity chromatography as previously described [44]. Briefly, 25 µL of plasma was diluted 7-fold with 1× phosphate-buffered saline (PBS in-house prepared), filtered through a water-wettable polytetrafluoroethylene (wwPTFE) filter plate with 0.45-µm pore size (Pall corporation, New York, NY, USA), and applied to a CIM^®^ r-Protein G LLD 0.05 mL 96-well monolithic plate (Sartorius BIA Separations, Ajdovščina, Slovenia). The plate was washed six times with 250 µL of 1× PBS (0.25 M sodium chloride (NaCl)), and bound IgG was eluted with 250 µL of 0.1 M formic acid (Merck, Darmstadt, Germany). Eluates were immediately neutralized with ammonium bicarbonate buffer (Sigma-Aldrich, St. Louis, MO, USA), and an aliquot of 20 µL was dried down in a vacuum centrifuge for subsequent processing.

### 2.4. Glycan Release and Labelling

Dried IgG samples were sequentially treated with 1.66× PBS, 0.5% sodium dodecyl sulphate (SDS; Sigma-Aldrich), and 2% Igepal (Sigma-Aldrich) to denature the IgG. N-glycans were released by incubation with 1.2U of Peptide:N-glycosidase F (PNGase F; (Promega, Madison, WI, USA) at 37 °C for 3 h. Released N-glycans were fluorescently labelled by mixing 8-aminopyrene-1,3,6-trisulfonic acid (APTS; Synchem, Felsberg, Germany) with 2-picoline borane (Sigma-Aldrich) and incubating the reaction at 37 °C for 16 h. The labelling reaction was quenched by adding 80% acetonitrile (ACN, Carlo Erba, Milan, Italy). Cleanup of APTS-labelled N-glycans was performed by solid-phase extraction using Bio-Gel P-10 as a hydrophilic stationary phase. The entire reaction mixture was transferred to a filter plate containing Bio-Gel P polyacrylamide gel (Bio-Gel P-10) and excess dye and reducing agent were removed by five washes with 80% ACN/100 mM triethylamine (Sigma-Aldrich), followed by three washes with 80% ACN. Labelled IgG N-glycans were eluted in ultrapure water for downstream analysis.

### 2.5. CGE-LIF

Labelled IgG N-glycans were analyzed by capillary gel electrophoresis with laser-induced fluorescence (CGE-LIF) as previously described [45]. Briefly, 3 µL of purified IgG N-glycans were mixed with 7 µL of Hi-Di Formamide and analyzed using an ABI3500 Genetic Analyzer (Thermo Fisher Scientific, Waltham, MA, USA) equipped with a 50-cm long 8-capillary array containing performance optimized polymer-7 as the separation matrix. Run parameters were set as follows: run time 1000 s, injection time 12 s, injection voltage 15 kV, run voltage 15 kV, and oven temperature 60 °C. Obtained electropherograms were manually integrated into 27 N-glycan peaks using Empower 3 software (Waters, Milford, MA, USA).

### 2.6. Statistical Analysis

To remove experimental variation, normalization and batch correction were performed on the CGE-LIF N-glycan data. Individual N-glycan peaks were expressed as a percentage of the total integrated area (total-area normalization) to ensure comparability across samples. Prior to batch correction, normalized measurements were log-transformed to address right-skewness and the multiplicative nature of technical variation. Batch effects (plate-to-plate variation) were modeled as a covariate and removed using the combating batch effects when combining batches (ComBat)method implemented in the R package (version 4.2.3).

From 27 directly measured IgG N-glycan peaks, six derived N-glycan traits were calculated based on groups of N-glycans sharing specific structural features (G0—agalactosylation, G1—monogalactosylation, G2—digalactosylation, S—sialylation, CF—core-fucosylation, and B—the presence of bisecting N-acetylglucosamine).

Before downstream analysis, all derived N-glycan traits were transformed to a standard normal distribution (mean = 0, SD = 1) using inverse rank-transformation to normality (transform function, GenABEL R package 1.8.0). This standardization ensures that the estimated effect sizes of different N-glycan traits are directly comparable.

Associations between disease status and derived N-glycan traits were evaluated using a general linear model, with age and sex included as fixed-effect covariates. For intracase analyses, associations between derived N-glycan traits and clinicopathological features (including molecular subtypes, IPI, aaIPI, R-IPI, NCCN-IPI, CNS-IPI, and ICPS risk scores) were examined using regression models adjusted for age and sex, with the derived N-glycan trait treated as the dependent variable.

Survival outcomes, including overall survival (OS), progression-free survival (PFS), and the risk of relapse, were analyzed using multivariable Cox proportional hazards models, adjusting for age and sex. OS was defined as the time from diagnosis to death from any cause, while PFS was defined as the time from diagnosis to disease progression, relapse, or death from any cause, whichever occurred first. Survivors or those lost to follow-up were censored at the time of last clinical contact (June 2025). Multiple testing was accounted for using the Benjamini–Hochberg False Discovery Rate (FDR) procedure, with an adjusted *p* < 0.05 considered statistically significant. Data were analyzed and visualized using R (version 4.2.3).

## 3. Results

### 3.1. IgG N-Glycome Composition in DLBCL, NOS Patients

This study included 54 patients with pathohistologically confirmed DLBCL, NOS and 54 age- and sex-matched healthy controls. The patient cohort exhibited an aggressive clinical profile, with a high prevalence of advanced-stage disease (76%) and extranodal involvement (74%) (Table 1). Furthermore, B-symptoms were present in 55% of patients, indicating a pronounced baseline systemic inflammatory state. All plasma samples were collected from treatment-naïve patients prior to the initiation of first-line therapy.

The composition of the IgG N-glycome was analyzed using CGE-LIF, which resolved the total IgG N-glycans into 27 distinct electrophoretic peaks (Figure 1). Because many of these individual structures share common structural features (e.g., core-fucose, galactose, and sialic acid moieties), six derived traits were calculated from the abundances of the 27 direct measurements (Supplementary Table S2). These derived N-glycan traits (G0, G1, G2, S, CF, and B) represent composite glycosylation features and provide a more comprehensive overview of the glycome.

Significant differences in the derived IgG N-glycan traits were observed between DLBCL, NOS patients and healthy controls (Figure 2; Supplementary Table S3). In general, patients exhibited a pro-inflammatory glycan signature characterized by a reduction in galactosylated and sialylated structures. Specifically, the abundance of agalactosylated N-glycans was significantly higher in patients (G0: effect = 0.452, *P*_adj_ = 1.35 × 10^−2^). Conversely, the abundances of digalactosylated (G2: effect = −0.434, *P*_adj_ = 1.35 × 10^−2^) and sialylated (S: effect = −0.552, *P*_adj_ = 1.35 × 10^−2^) structures were significantly decreased in the patient cohort. Furthermore, the abundance of core-fucosylated IgG N-glycans was elevated in DLBCL, NOS patients, although this trend did not reach the threshold for statistical significance. Similarly, a non-significant decrease was observed in the abundance of bisected N-glycans structures among the patient group.

Finally, we evaluated differences between the GCB and non-GCB subtypes as determined by the Hans algorithm. We observed a significantly lower abundance of monogalactosylated N-glycans in the non-GCB subtype compared to the GCB subtype (G1: effect = −0.272, *P*_adj_ = 3.91 × 10^−2^) (Supplementary Figure S1; Supplementary Table S4).

### 3.2. Association of IgG N-Glycome with Prognostic Indices

Next, we evaluated the association between the IgG N-glycome and risk groups derived from established clinical prognostic indices, including the IPI, aaIPI (stratified for patients < 60 and ≥60 years of age), R-IPI, NCCN-IPI, CNS-IPI, as well as the inflammation-based ICPS. A significant association was observed across the different indices, where higher-risk categories, comprising a greater number of clinical risk factors, were consistently associated with decreased IgG galactosylation (Figure 3; Supplementary Figure S1; Supplementary Table S4). Specifically, a significant positive association was observed between IPI risk groups and agalactosylation (G0: effect = 0.334, *P*_adj_ = 1.05 × 10^−3^), while monogalactosylation (G1: effect = −0.268, *P*_adj_ = 2.08 × 10^−2^) and digalactosylation (G2: effect = −0.363, *P*_adj_ = 3.63 × 10^−4^) were strongly inversely correlated (Figure 3). Similar robust associations were confirmed for the R-IPI (G0: effect = 0.316, *P*_adj_ = 1.17 × 10^−3^; G2: effect = −0.341, *P*_adj_ = 6.54 × 10^−4^), NCCN-IPI (G0: effect = 0.360, *P*_adj_ = 6.73 × 10^−3^; G2: effect = −0.380, *P*_adj_ = 4.15 × 10^−3^), and CNS-IPI risk groups (G0: effect = 0.282, *P*_adj_ = 5.22 × 10^−3^; G2: effect = −0.319, *P*_adj_ = 1.14 × 10^−3^), all of which were significantly associated with both increased G0 and decreased G2. In addition, equivalent associations were observed for the ICPS risk groups (G0: effect = 0.307, *P*_adj_ = 1.05 × 10^−3^; G2: effect = −0.351, *P*_adj_ = 2.51 × 10^−4^), highlighting a strong link between systemic inflammatory burden and the loss of IgG galactosylation.

### 3.3. The Prognostic Value of the IgG N-Glycome

Given that the IgG N-glycome was strongly associated with a DLBCL, NOS diagnosis, we explored its prognostic value regarding clinical outcomes. Among the 54 patients in the cohort, 20 (37%) experienced a relapse, and 21 (39%) deaths occurred during the follow-up period. Multivariable analysis using the Cox proportional hazards model identified IgG galactosylation and sialylation as significant predictors of clinical outcomes, specifically regarding the risk of relapse and PFS (Figure 4, Supplementary Figure S2; Supplementary Table S5). In particular, an increased abundance of agalactosylated N-glycans was significantly associated with a shorter PFS (G0: effect = 0.581, *P*_adj_ = 4.82 × 10^−2^). Conversely, a decrease in the abundance of sialylated structures was strongly associated with both an increased risk of disease progression and relapse. Specifically, reduced sialylation was a significant predictor of shorter PFS (S: effect = −0.733, *P*_adj_ = 1.12 × 10^−2^) and was also significantly associated with a higher probability of relapse (S: effect = −0.764, *P*_adj_ = 3.09 × 10^−2^).

## 4. Discussion

In this study, we comprehensively characterized previously unexplored plasma IgG N-glycosylation in patients with DLBCL, NOS and demonstrated that the plasma IgG N-glycome is markedly altered compared with healthy controls. These alterations were further associated with molecular subtype, established clinical prognostic indices, and survival outcomes, highlighting the potential of plasma IgG N-glycosylation as a biomarker of disease biology and prognosis.

Consistent with previous observations in chronic inflammatory states and malignant conditions [28,47], DLBCL, NOS patients exhibited a pronounced shift toward a pro-inflammatory IgG N-glycosylation profile characterized by increased agalactosylation (G0) and reduced digalactosylation (G2) and sialylation (S). These glycosylation features are known to regulate antibody effector functions by influencing Fc conformation, Fcγ receptor interactions, and complement activation, thereby shaping downstream inflammatory and immune responses [26,48]. In particular, reduced galactosylation and sialylation have been consistently associated with a pro-inflammatory immune phenotype, whereas Fc sialylation is widely recognized to promote immunoregulatory and anti-inflammatory responses [26,28,48]. Given that more than half of the patients in our cohort presented with B-symptoms and a high proportion had advanced-stage disease, the observed glycosylation profile is consistent with the systemic immune activation and chronic inflammation that accompany aggressive lymphoma biology [4]. Furthermore, these findings suggest that DLBCL, NOS is accompanied by broader dysregulation of the humoral immune response reflected in the plasma IgG N-glycome.

Beyond the differences observed between patients and healthy controls, we identified significantly lower IgG monogalactosylation (G1) in the non-GCB subtype compared with GCB DLBCL. Although the biological basis of this observation remains unclear, the non-GCB subtype is characterized by chronic active BCR signaling and constitutive NF-κB activation, molecular features associated with inferior clinical outcomes [4,16]. Conversely, GCB-DLBCL frequently acquires tumor-specific BCR N-glycosylation that promotes lectin-mediated signaling and lymphoma cell survival [36,37]. Although these studies examined glycosylation of the malignant BCR rather than plasma IgG, they support the broader concept that glycosylation is an important component of DLBCL biology. Our findings further suggest that systemic IgG N-glycosylation may reflect differences in host immune responses accompanying distinct biological subtypes of DLBCL, NOS.

One of the most striking findings of our study was the highly consistent association between IgG galactosylation and multiple established prognostic scoring systems. Across the IPI, R-IPI, NCCN-IPI, CNS-IPI, and ICPS, increasing clinical risk was consistently associated with progressive loss of galactosylation, reflected by reduced digalactosylation (G2) and accumulation of agalactosylated glycans (G0). The remarkable consistency of these associations across multiple established and complementary prognostic models suggests that alterations in IgG galactosylation reflect a common biological process associated with disease aggressiveness rather than being driven by any single clinical parameter. Because reduced IgG galactosylation is a well-established hallmark of chronic inflammation and systemic immune activation [28], these findings are consistent with the hypothesis that patients with more aggressive disease exhibit greater immune dysregulation. Furthermore, the observed association with the ICPS, an inflammation-based prognostic score, provides additional support for a close relationship between plasma IgG glycosylation and the inflammatory milieu in DLBCL, NOS.

The prognostic relevance of IgG glycosylation was further supported by survival analyses. Increased agalactosylation was associated with shorter PFS, whereas reduced sialylation predicted both inferior PFS and an increased risk of relapse. These findings suggest that alterations in IgG glycosylation are associated not only with baseline disease characteristics but also with subsequent clinical outcomes. Given the established immunoregulatory role of Fc sialylation, reduced IgG sialylation may reflect persistent systemic inflammation and impaired immune homeostasis accompanying more aggressive disease. Although recent experimental studies have demonstrated that N-linked glycosylation is essential for oncogenic BCR signaling and lymphoma cell survival in both GCB and non-GCB DLBCL [49], these studies address tumor-intrinsic glycosylation and should be distinguished from the systemic plasma IgG N-glycosylation assessed here. The cellular origin of the plasma IgG glycosylation changes observed in our study cannot be determined from the present data. Given that our analysis assessed the total circulating IgG N-glycome rather than glycosylation of the malignant B-cell clone, these alterations may predominantly reflect changes in the systemic humoral immune response accompanying DLBCL. However, potential contributions of lymphoma-associated changes in B-cell biology and the tumor microenvironment cannot be excluded. Therefore, whether altered plasma IgG N-glycosylation contributes functionally to disease progression or primarily represents a correlative biomarker of the host response to lymphoma remains to be determined. Future mechanistic studies will be required to establish the cellular origin and functional consequences of these alterations. Profiling the secretome and glycosylation characteristics of DLBCL-derived B-cell models could help determine whether malignant cells contribute to the observed glycosylation alterations, while functional studies using patient-derived purified IgG, including antibody-dependent cellular cytotoxicity (ADCC) and complement-dependent cytotoxicity (CDC) assays, could assess whether the observed glycosylation differences translate into altered antibody effector functions.

This study has several strengths. To our knowledge, it represents the first comprehensive characterization of plasma IgG N-glycosylation in DLBCL, NOS and its relationship with molecular subtype, established prognostic indices, and clinical outcomes. Additional strengths include the analysis of treatment-naïve patients, the inclusion of age- and sex-matched healthy controls, detailed clinical characterization, and the application of high-resolution CGE-LIF analysis for quantitative IgG N-glycan profiling. Nevertheless, several limitations should be acknowledged. First, the relatively modest sample size and exploratory nature of this cohort limited statistical power, particularly for subgroup and survival analyses, and may have precluded detection of weaker associations. Accordingly, the observed associations should be considered hypothesis-generating and require independent validation in larger, prospectively collected cohorts. Second, IgG glycosylation was assessed at a single pre-treatment time point, preventing evaluation of dynamic glycosylation changes during treatment and disease progression. Third, COO classification was based on the Hans immunohistochemical algorithm rather than gene expression profiling (GEP). Although the Hans algorithm represents a practical and widely used approach to COO classification in routine clinical practice, and its use was considered acceptable under the 2016 WHO classification applied in this study, it remains an imperfect surrogate for GEP. In a cohort of 359 patients with DLBCL, concordance between the Hans algorithm and the Lymph2Cx GEP assay was 72%, and, unlike GEP, the Hans algorithm does not identify an unclassified molecular subgroup [50]. Furthermore, the prognostic performance of Hans-based classification has been inconsistent. A meta-analysis of 1970 patients found no significant difference in OS between Hans-defined GCB and non-GCB subtypes, although a significant difference in PFS was observed [51]. Therefore, some degree of COO misclassification in our study cannot be excluded. Finally, the observational design precludes conclusions regarding causal relationships between altered IgG glycosylation and disease biology or clinical outcomes. External validation in independent cohorts will therefore be essential before these findings can be translated into clinical practice.

In conclusion, this exploratory study demonstrates for the first time that plasma IgG N-glycosylation is profoundly altered in patients with DLBCL, NOS and is associated with molecular subtype, established prognostic indices, and clinically relevant outcomes, particularly PFS and relapse. These findings identify the plasma IgG N-glycome as a potential correlative biomarker of DLBCL disease characteristics and clinical outcome but do not establish a causal role for altered IgG glycosylation in lymphoma progression. Larger prospective, longitudinal studies with independent validation are required to determine the robustness and incremental prognostic value of these associations and to establish whether plasma IgG N-glycosylation has clinical utility for risk stratification or disease monitoring.

## Figures and Tables

**Figure 1:**
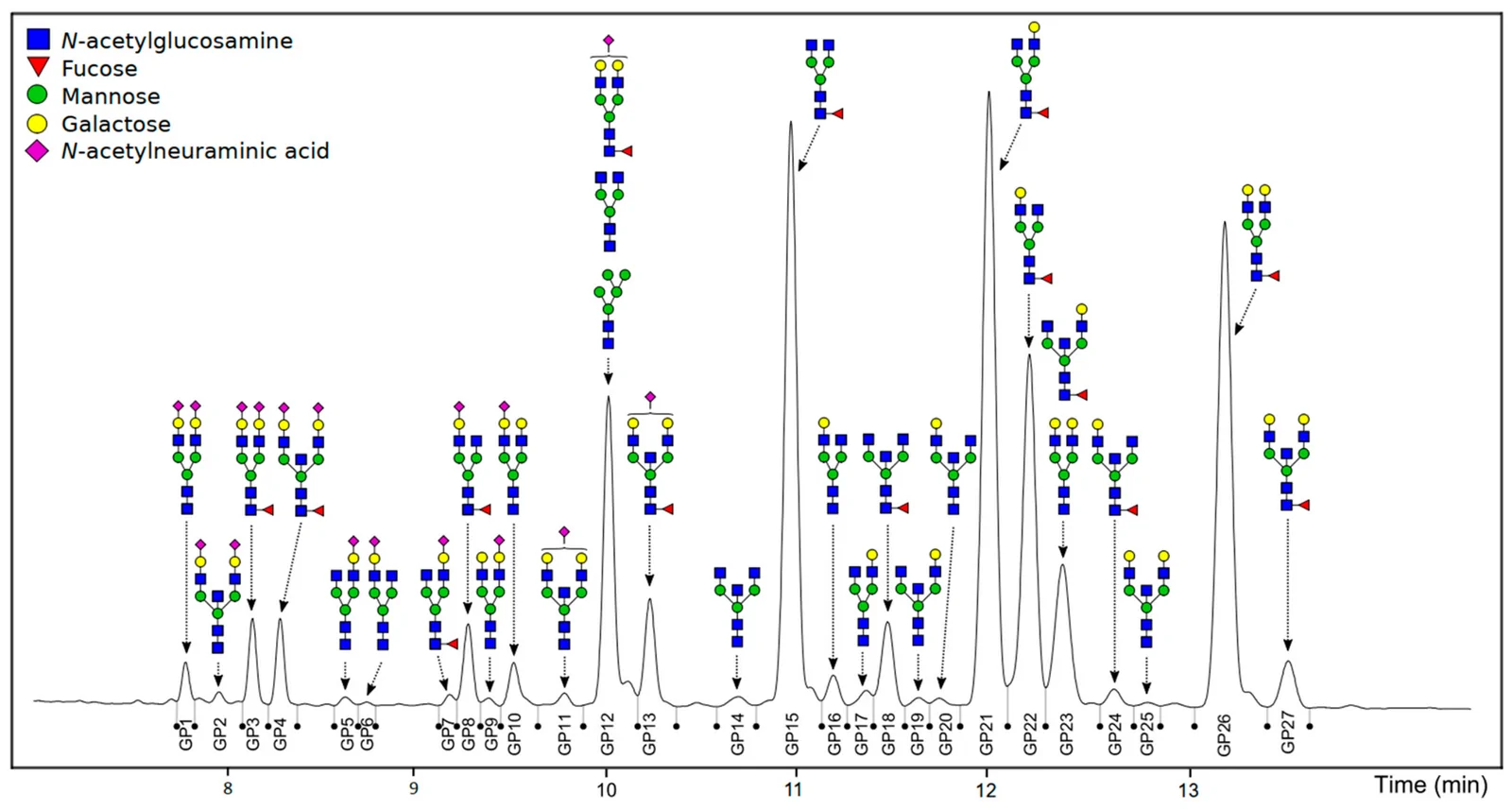
Representative electropherogram of plasma IgG N-glycans. Total IgG was isolated by Protein G affinity chromatography, and N-glycans were enzymatically released using PNGase F, fluorescently labelled with 8-aminopyrene-1,2,6-trisulfonic acid (APTS), purified, and separated by capillary gel electrophoresis with laser-induced fluorescence (CGE-LIF). Electropherograms were integrated into 27 glycan peaks, each containing one or more N-glycan structures assigned previously using GlyXTool [46]. Glycan structures are shown using Symbol Nomenclature for Glycans (SNFG): blue square, *N*-acetylglucosamine; red triangle, fucose; green circle, mannose; yellow circle, galactose; purple diamond, *N*-acetylneuraminic acid.

**Figure 2:**
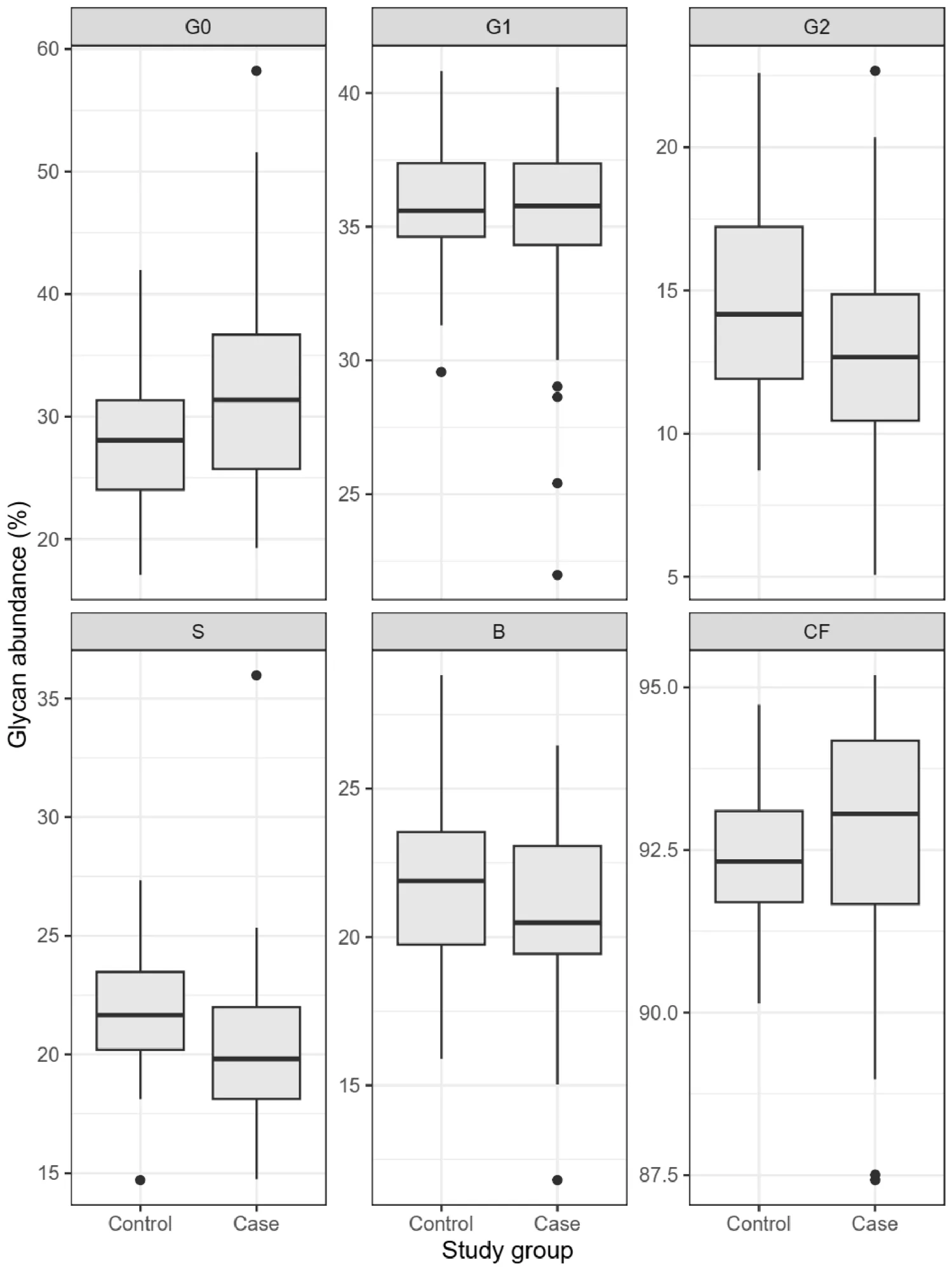
Differences in IgG N-glycome between healthy controls and patients with DLBCL-NOS. Relative abundances of calculated derived IgG glycosylation traits are presented as box plots representing the 25th and 75th percentile. Lines inside the boxes represent the median. Lines outside the boxes represent the 10th and 90th percentiles. Individual points beyond the whiskers represent outliers. G0—agalactosylated, G1—monogalactosylated, G2—digalactosylated, S—sialylated, B—bisected, and CF—core-fucosylated glycans.

**Figure 3:**
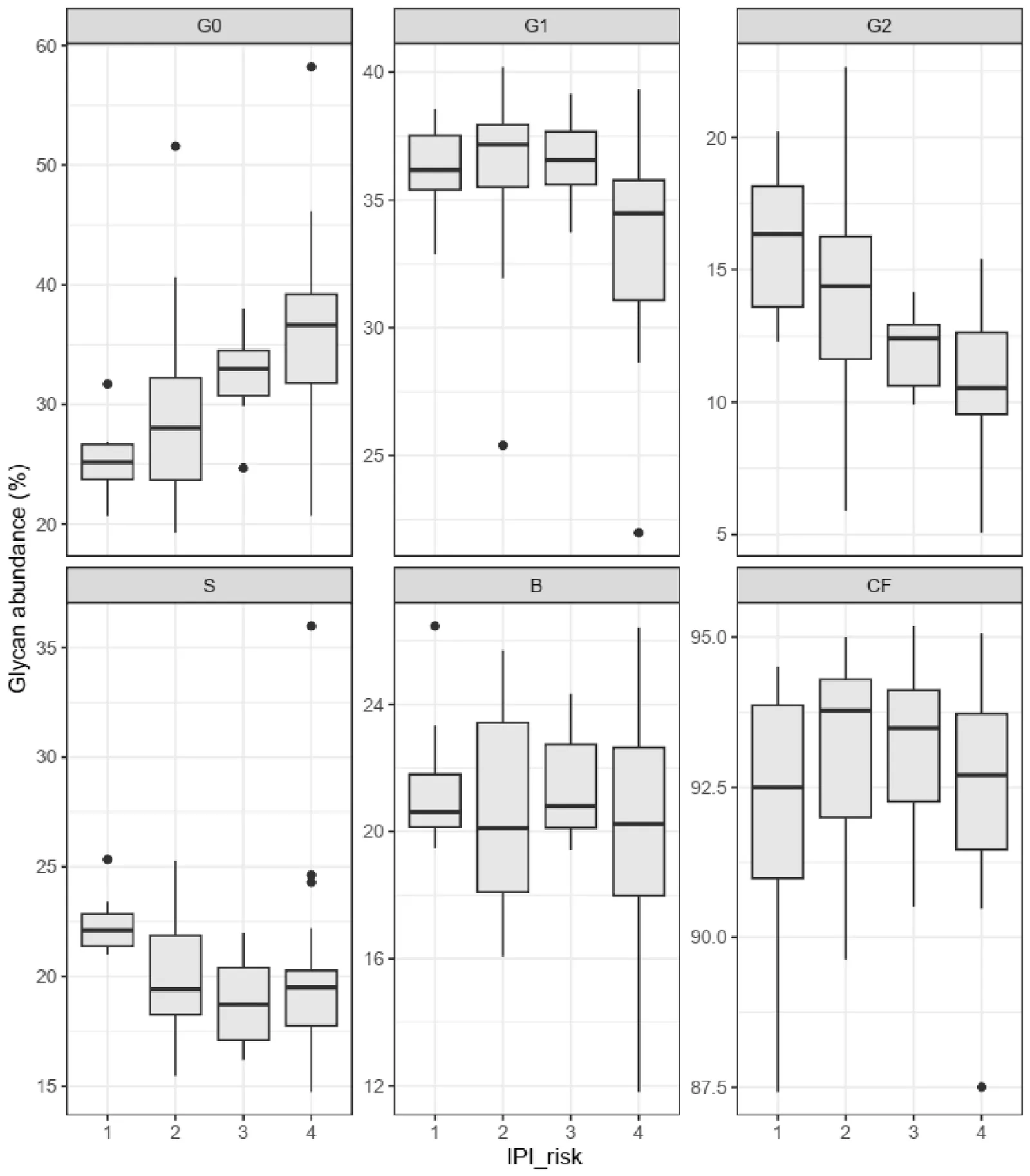
Plasma IgG N-glycosylation traits across International Prognostic Index (IPI) risk categories in patients with DLBCL-NOS. Relative abundances of calculated derived IgG glycosylation traits across increasing IPI risk categories (1, low; 2, low-intermediate; 3, high-intermediate; 4, high) are presented as box plots representing the 25th and 75th percentile. Lines inside the boxes represent the median. Lines outside the boxes represent the 10th and 90th percentiles. Individual points beyond the whiskers represent outliers. G0—agalactosylated, G1—monogalactosylated, G2—digalactosylated, S—sialylated, B—bisected, and CF—core-fucosylated glycans. Higher IPI risk was significantly associated with increased G0 and decreased G2 after adjustment for age and sex and correction for multiple testing.

**Figure 4:**
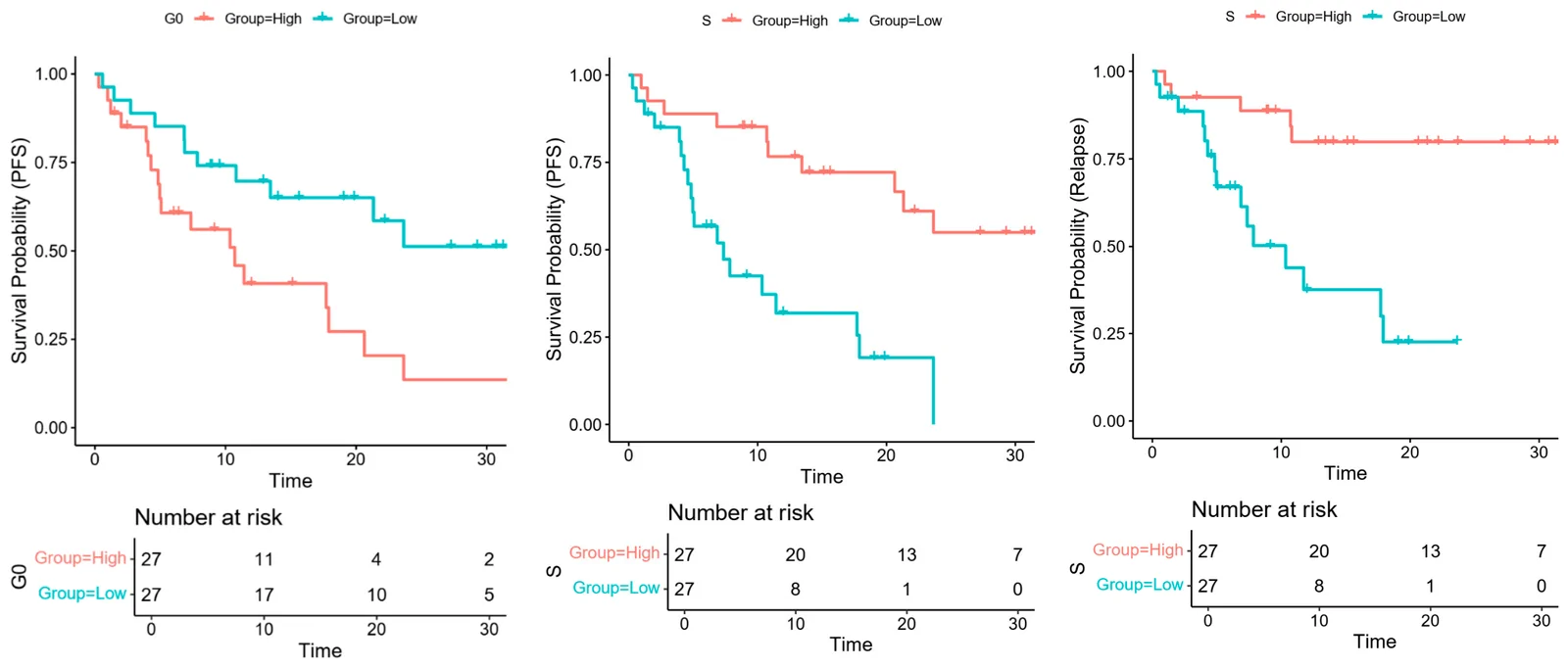
Kaplan–Meier analysis of progression-free survival (PFS) and the risk of relapse according to IgG N-glycosylation in DLBCL, NOS patients. Patients were stratified into high (red line) and low (turquoise line) groups according to the median values of the derived IgG N-glycosylation traits. Only statistically significant findings are shown. G0—agalactosylated, S—sialylated glycans.

**Table 1:** Demographic characteristics of healthy controls and clinicopathological features of DLBCL, NOS patients.

Characteristics	Values
DLBCL, NOS patients (*n* = 54)	
Demographics	
Age (years), median (IQR)	70 (60–75)
Sex (female/male), *n* (%)	31 (57%)/23 (43%)
Molecular subtype ^1^	
GCB, *n* (%)	31 (57%)
non-GCB, *n* (%)	23 (43%)
Clinical presentation	
Clinical stage (Ann Arbor classification), *n*(%)	Stages I or II: 13 (24%) Stages III or IV: 41 (76%)
Extranodal involvement, *n* (%)	40 (74%)
Presence of “B-symptoms”, *n*(%)	29 (55%)
Bulky disease ^2^, *n*(%)	21 (39%)
Prognostic risk groups, *n*(%)	
IPI risk group (Low/Low-Inter/High-Inter, High)	8 (15%)/18 (33%)/8 (15%)/20 (37%)
aaIPI-young risk group (<60 years) (Low/Low-Inter/High-Inter, High)	2 (12%)/6 (35%)/3 (18%)/6 (35%)
aaIPI-old risk group (≥60 years) (Low/Low-Inter/High-Inter, High)	3 (8%)/8 (22%)/12 (32%)/14 (38%)
R-IPI risk group (Very good/Good/Poor)	8 (15%)/18 (33%)/28 (52%)
NCCN-IPI risk group (Low/Low-Inter/High-Inter, High)	4 (7%)/16 (30%)/21 (39%)/13 (24%)
CNS-IPI risk group (Low/Inter/High)	9 (17%)/20 (37%)/25 (46%)
ICPS risk group (Low/Inter/High)	7 (13%)/13 (24%)/34 (63%)
Therapy, *n* (%)	
R-CHOP-21	29 (54%)
R-DA-EPOCH	13 (24%)
R-mini-CHOP-21	8 (15%)
Pola-R-CHP	4 (7%)
Outcomes	
Relapse	20 (37%)
Death	21 (39%)
Healthy controls (*n* = 54)	
Demographics	
Age (years), median (IQR)	70 (60, 75)
Sex (female/male), *n* (%)	31 (57%)/23 (43%)

^1^ Subtypes determined by the Hans immunohistochemical algorithm. ^2^ Bulky disease was defined as the presence of one or more lesions that were 7.5 cm or larger in greatest dimension.

## Data Availability

The raw data supporting the conclusions of this article are available at https://zenodo.org/me/uploads?q=&f=shared_with_me%3Afalse&l=list&p=1&s=10&sort=newest (accessed on 1 September 2026).

## Supplementary Materials

The following supporting information can be downloaded at: https://www.mdpi.com/article/10.3390/biomedicines14102150/s1, Figure S1: IgG N-glycosylation traits according to molecular subtype and clinical prognostic scores in DLBCL. Figure S2: Kaplan–Meier analyses of progression-free survival (PFS), relapse, and overall survival according to IgG N-glycosylation traits in DLBCL patients. Table S1: Laboratory parameters of DLBCL patients. Table S2: Description of derived IgG N-glycosylation traits calculated from 27 electrophoretic peaks (GP1-27). Table S3: Association of derived IgG N-glycosylation traits with DLBCL status. Table S4: Association of derived IgG N-glycosylation traits with molecular subtype and clinical prognostic indices in DLBCL patients. Table S5: Association of derived IgG N-glycosylation traits with clinical outcomes in DLBCL patients.
