# Supplementary material for: Plasma IgG N-Glycosylation as a Novel Biomarker of Disease Severity and Prognosis in Diffuse Large B-Cell Lymphoma, Not Otherwise Specified
Source: Biomedicines. 2026 Sep 23;14(10):2150. doi: 10.3390/biomedicines14102150 (PMC13701574; doi:10.3390/biomedicines14102150)
Supplement: Supplementary file 1 [file biomedicines-14-02150-s001.zip › Milunović biomedicine supplementary Figure S1.pdf]

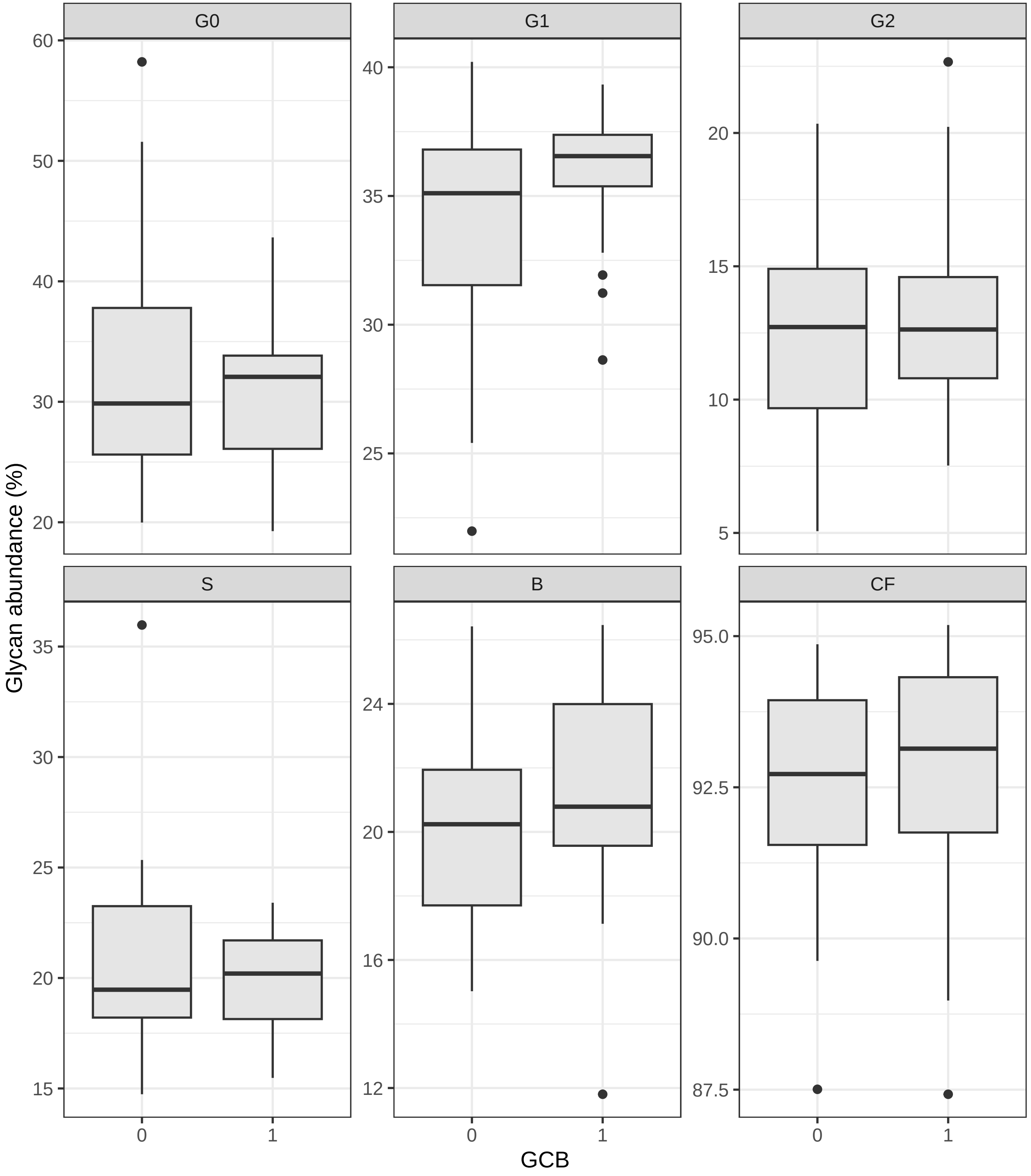
G0
G1
G2
S
B
CF
Glycan abundance (%)
GCB
60
50
40
30
20
25
35
15
10
5
95.0
92.5
90.0
87.5
24
16
12
0
1

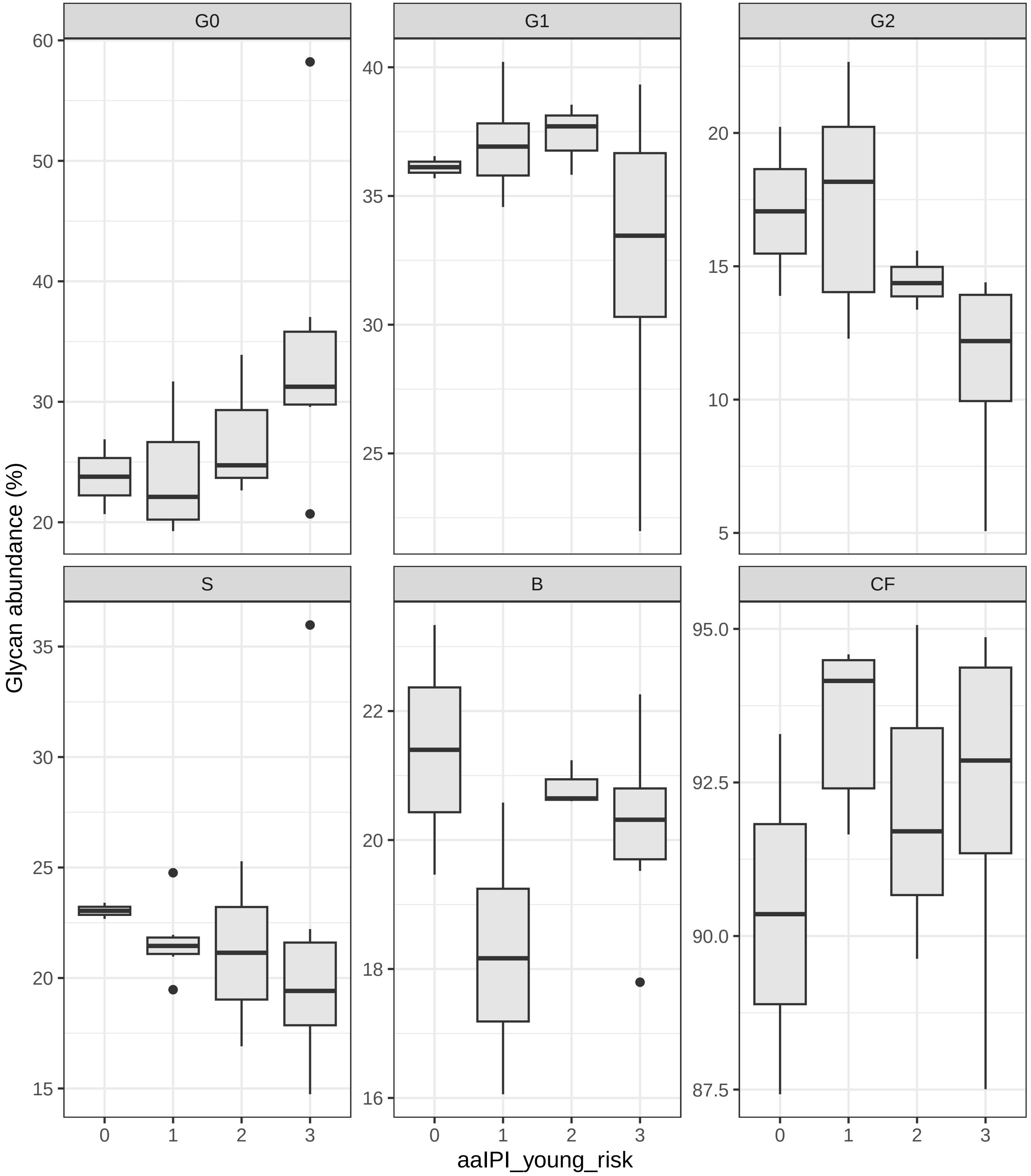
G0
G1
G2
S
B
CF
Glycan abundance (%)
aaIPI_young_risk
0
1
2
3
60
50
40
30
20
25
35
15
22
18
16
10
5
95.0
92.5
90.0
87.5

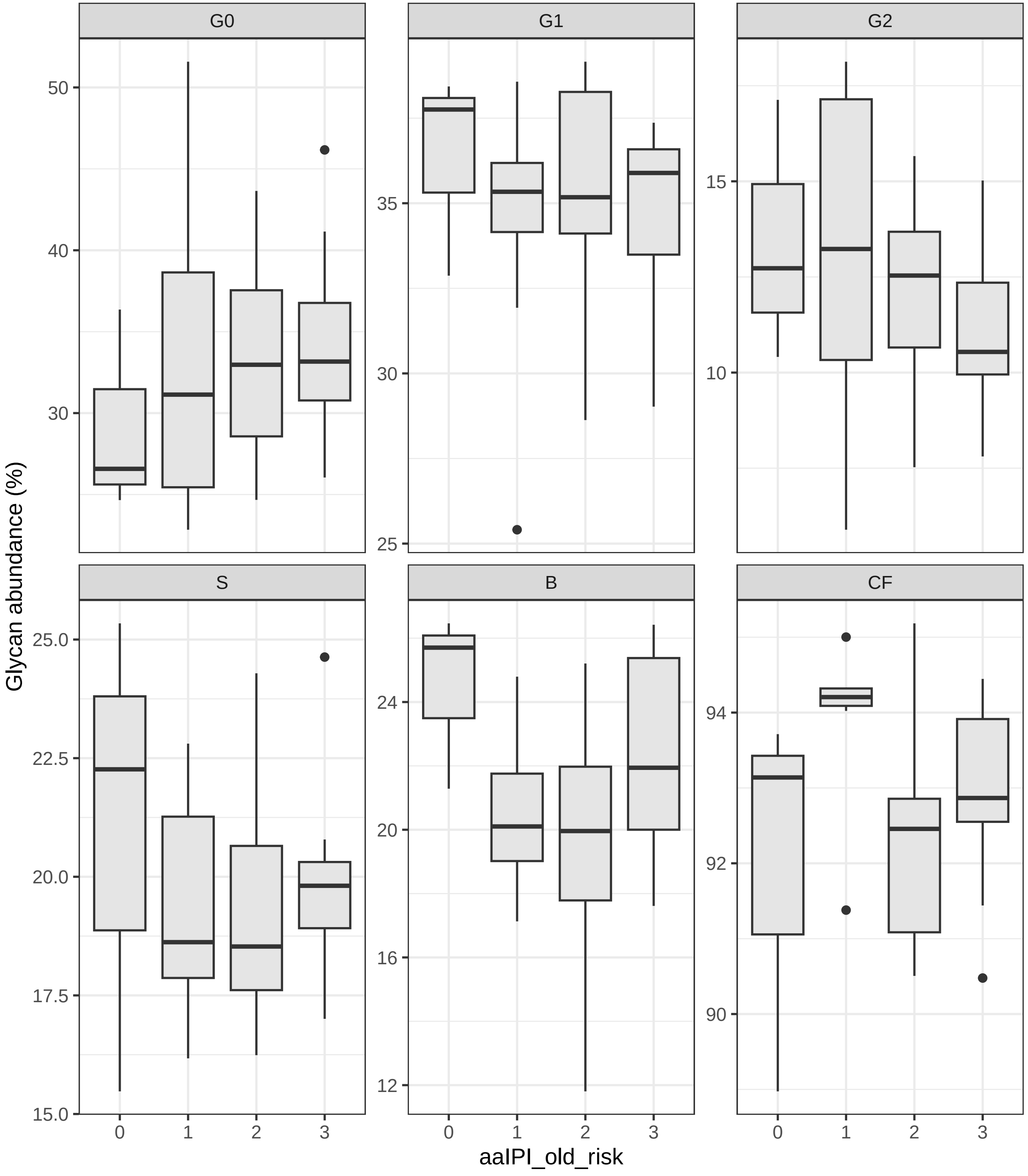
G0
G1
G2
S
B
CF
Glycan abundance (%)
aaIPI_old_risk
0
1
2
3
50
40
30
35
25
15
10
25.0
22.5
20.0
17.5
15.0
24
20
16
12
94
92
90

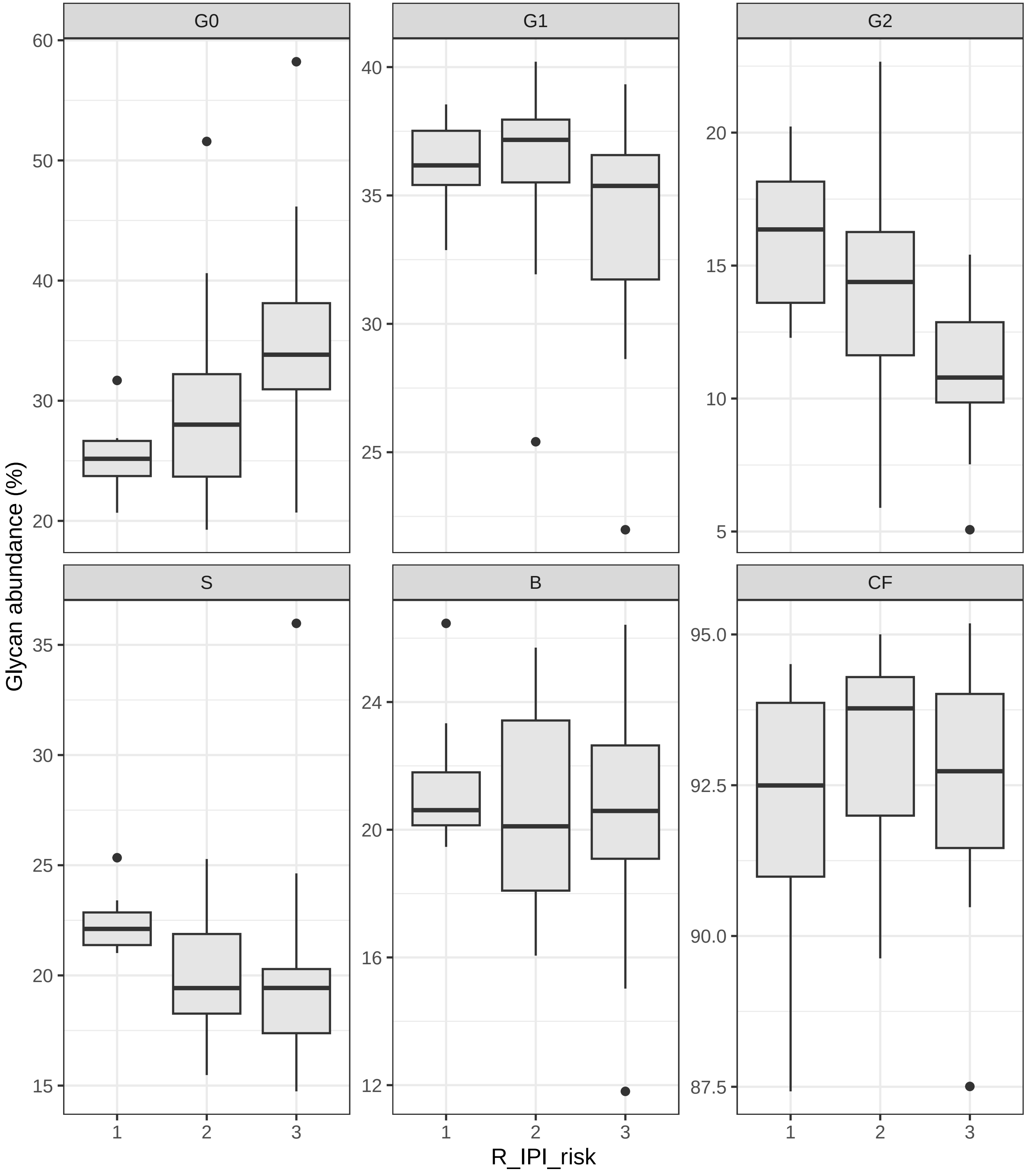

G0
G1
G2
S
B
CF
Glycan abundance (%)
R_IPI_risk
1
2
3
60
50
40
30
20
40
35
30
25
20
15
10
5
35
30
25
20
15
24
20
16
12
95.0
92.5
90.0
87.5

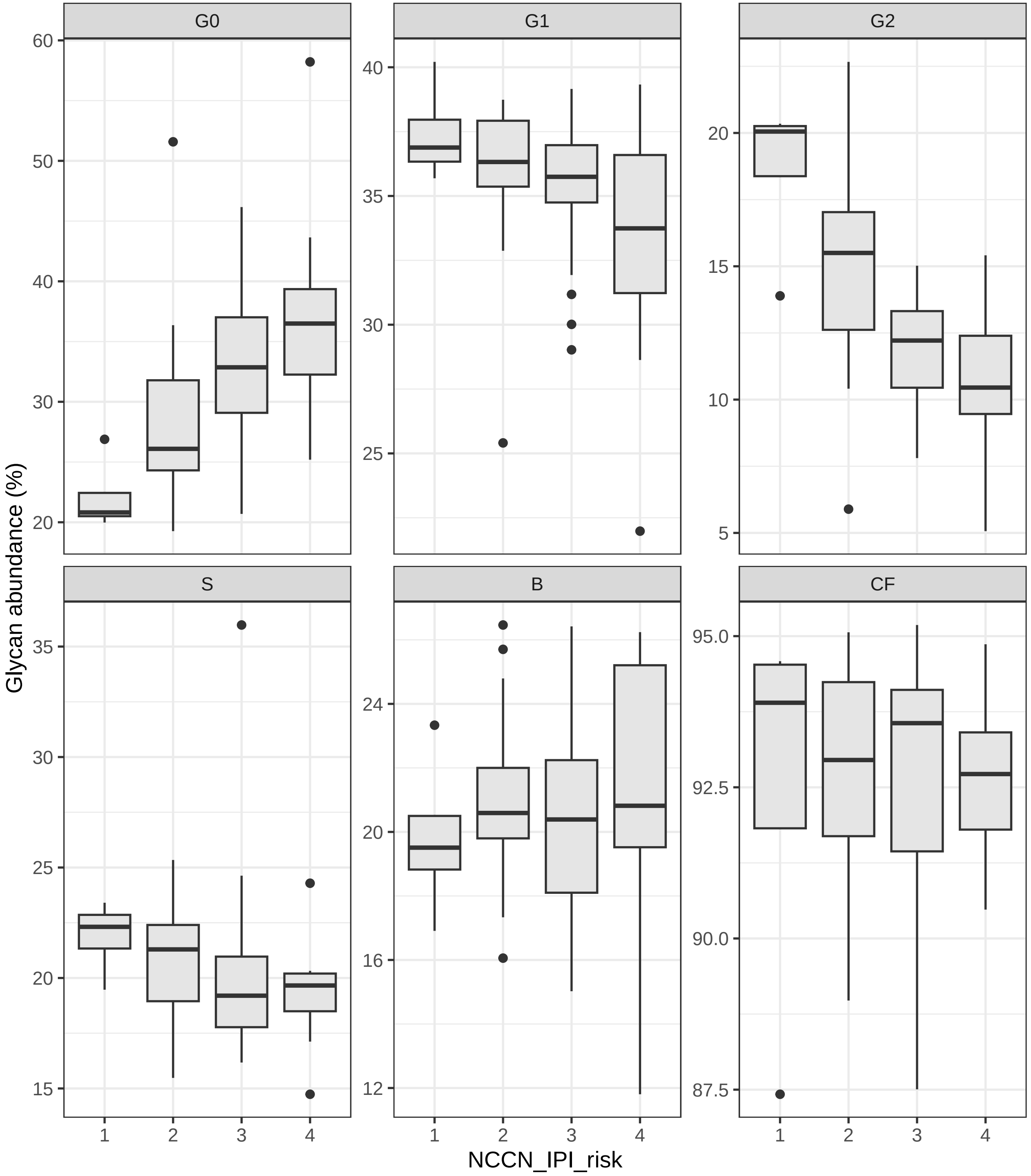

G0
G1
G2
S
B
CF
Glycan abundance (%)
NCCN_IPI_risk
60
50
40
30
20
35
25
15
40
35
30
25
20
15
10
5
24
20
16
12
95.0
92.5
90.0
87.5
1
2
3
4

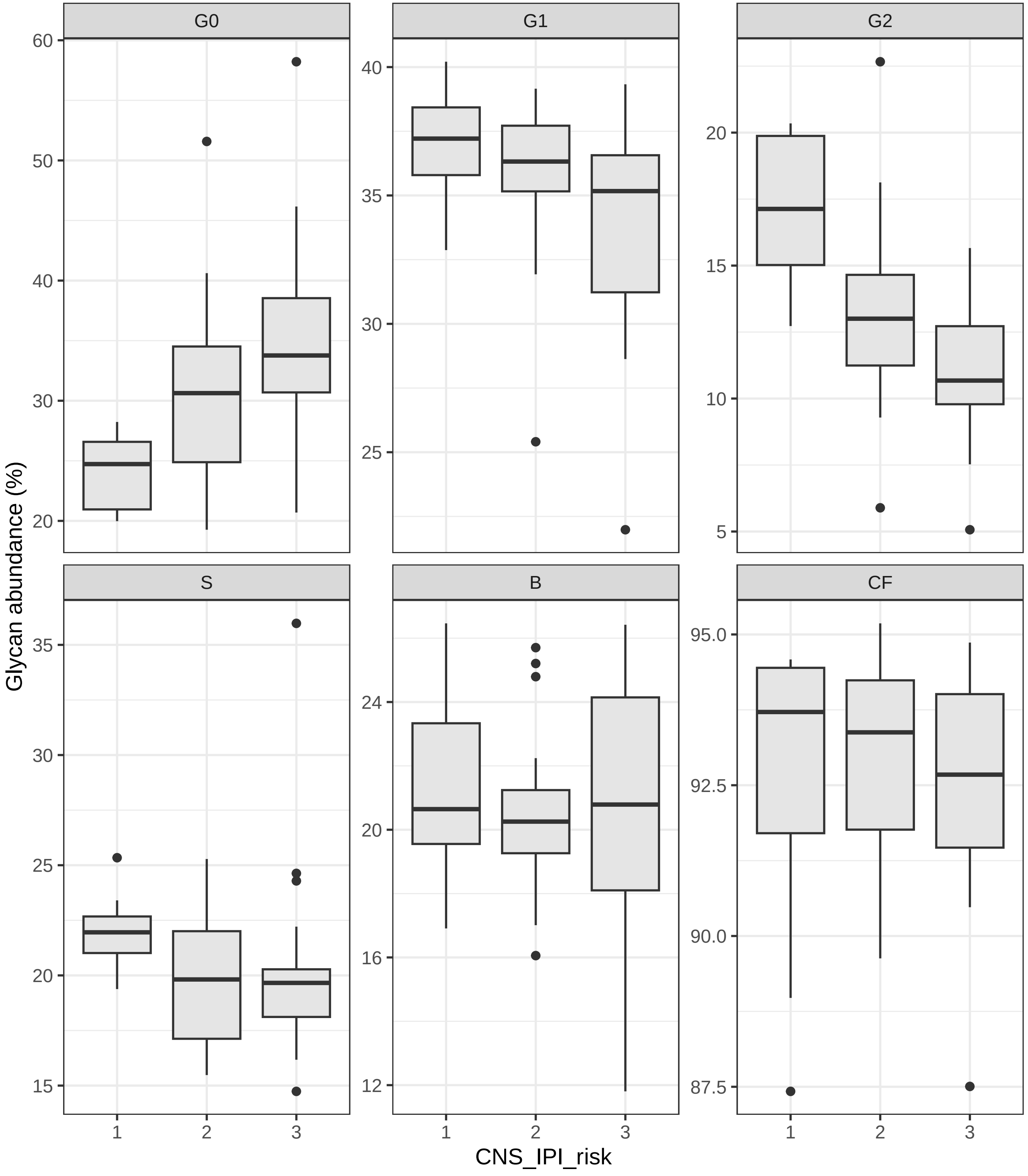
G0
G1
G2
S
B
CF
60
50
40
30
20
40
35
30
25
20
15
10
5
35
30
25
20
15
24
20
16
12
95.0
92.5
90.0
87.5
1
2
3
Glycan abundance (%)
CNS_IPI_risk

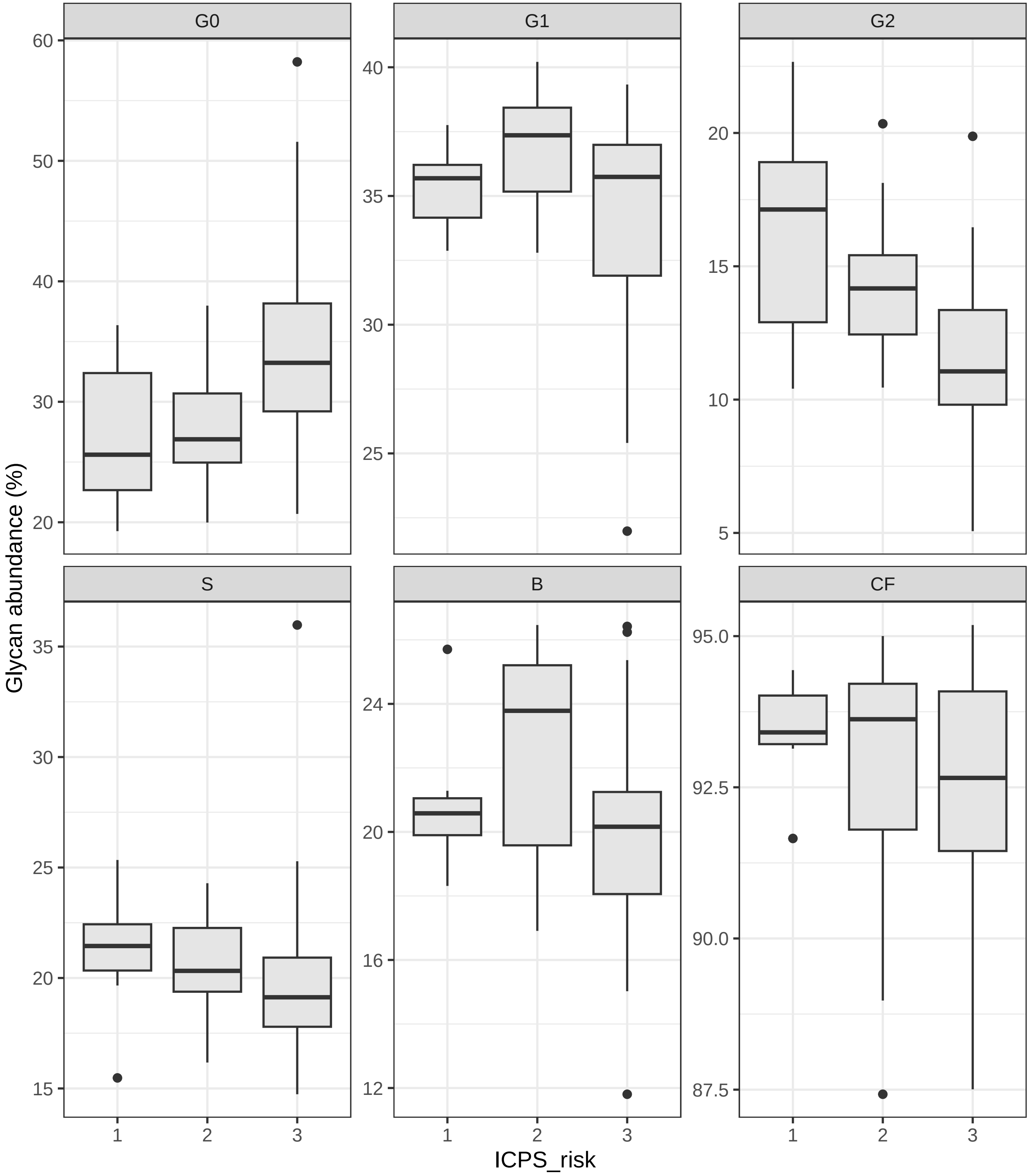

G0
G1
G2
S
B
CF
Glycan abundance (%)
ICPS_risk
1
2
3
60
50
40
30
20
40
35
30
25
20
15
10
5
35
30
25
20
15
24
20
16
12
95.0
92.5
90.0
87.5
