# Supplementary material for: Plasma IgG N-Glycosylation as a Novel Biomarker of Disease Severity and Prognosis in Diffuse Large B-Cell Lymphoma, Not Otherwise Specified
Source: Biomedicines. 2026 Sep 23;14(10):2150. doi: 10.3390/biomedicines14102150 (PMC13701574; doi:10.3390/biomedicines14102150)
Supplement: Supplementary file 1 [file biomedicines-14-02150-s001.zip › Milunović biomedicine supplementary Figure S2.pdf]

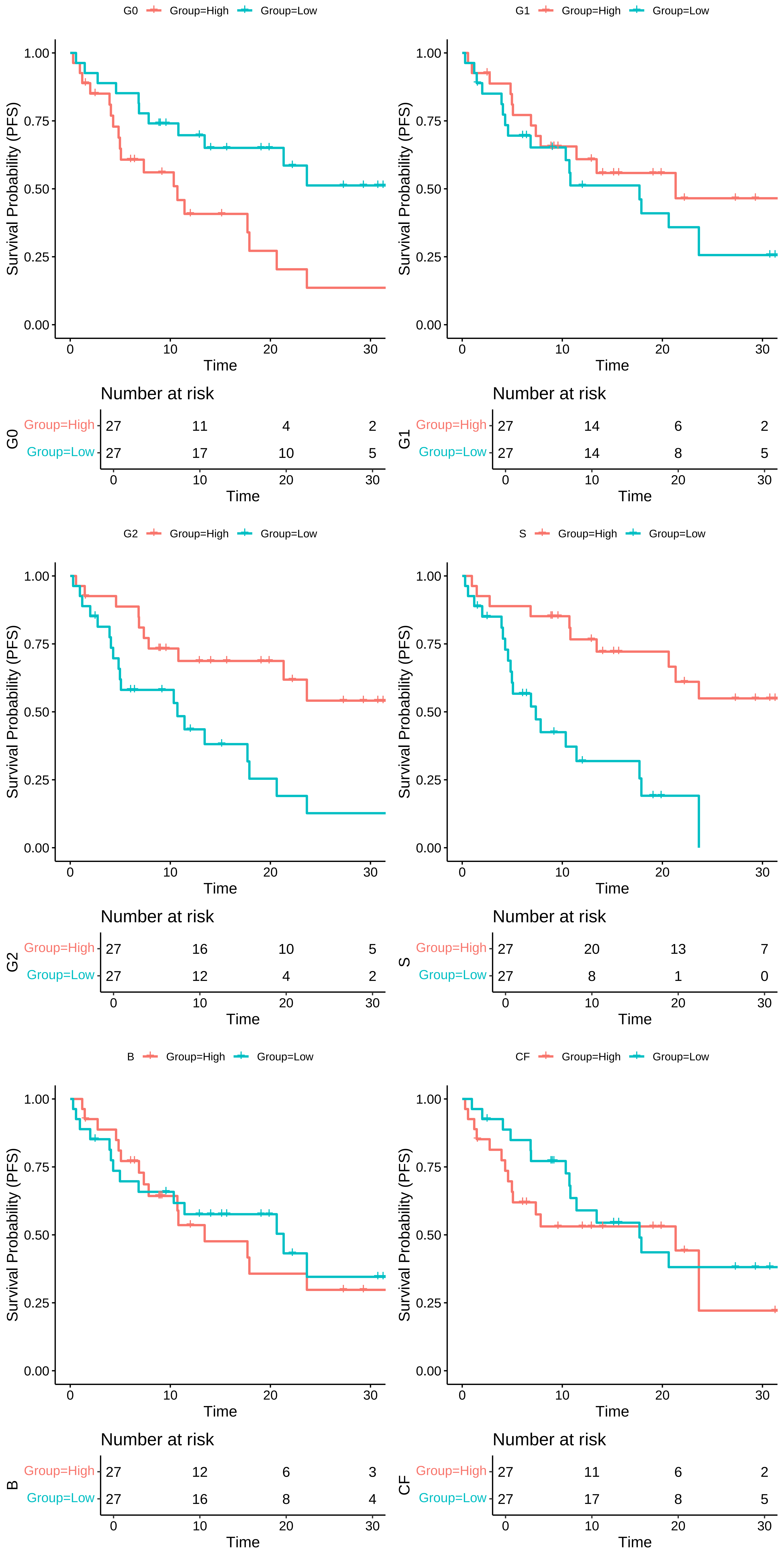
G0 Group=High Group=Low
Survival Probability (PFS)
1.00
0.75
0.50
0.25
0.00
0 10 20 30
Time
Number at risk
G0
Group=High 27 11 4 2
Group=Low 27 17 10 5
0 10 20 30
Time
G1 Group=High Group=Low
Survival Probability (PFS)
Time
Number at risk
G1
Group=High 27 14 6 2
Group=Low 27 14 8 5
G2 Group=High Group=Low
Survival Probability (PFS)
Time
Number at risk
G2
Group=High 27 16 10 5
Group=Low 27 12 4 2
S Group=High Group=Low
Survival Probability (PFS)
Time
Number at risk
S
Group=High 27 20 13 7
Group=Low 27 8 1 0
B Group=High Group=Low
Survival Probability (PFS)
Time
Number at risk
B
Group=High 27 12 6 3
Group=Low 27 16 8 4
CF Group=High Group=Low
Survival Probability (PFS)
Time
Number at risk
CF
Group=High 27 11 6 2
Group=Low 27 17 8 5

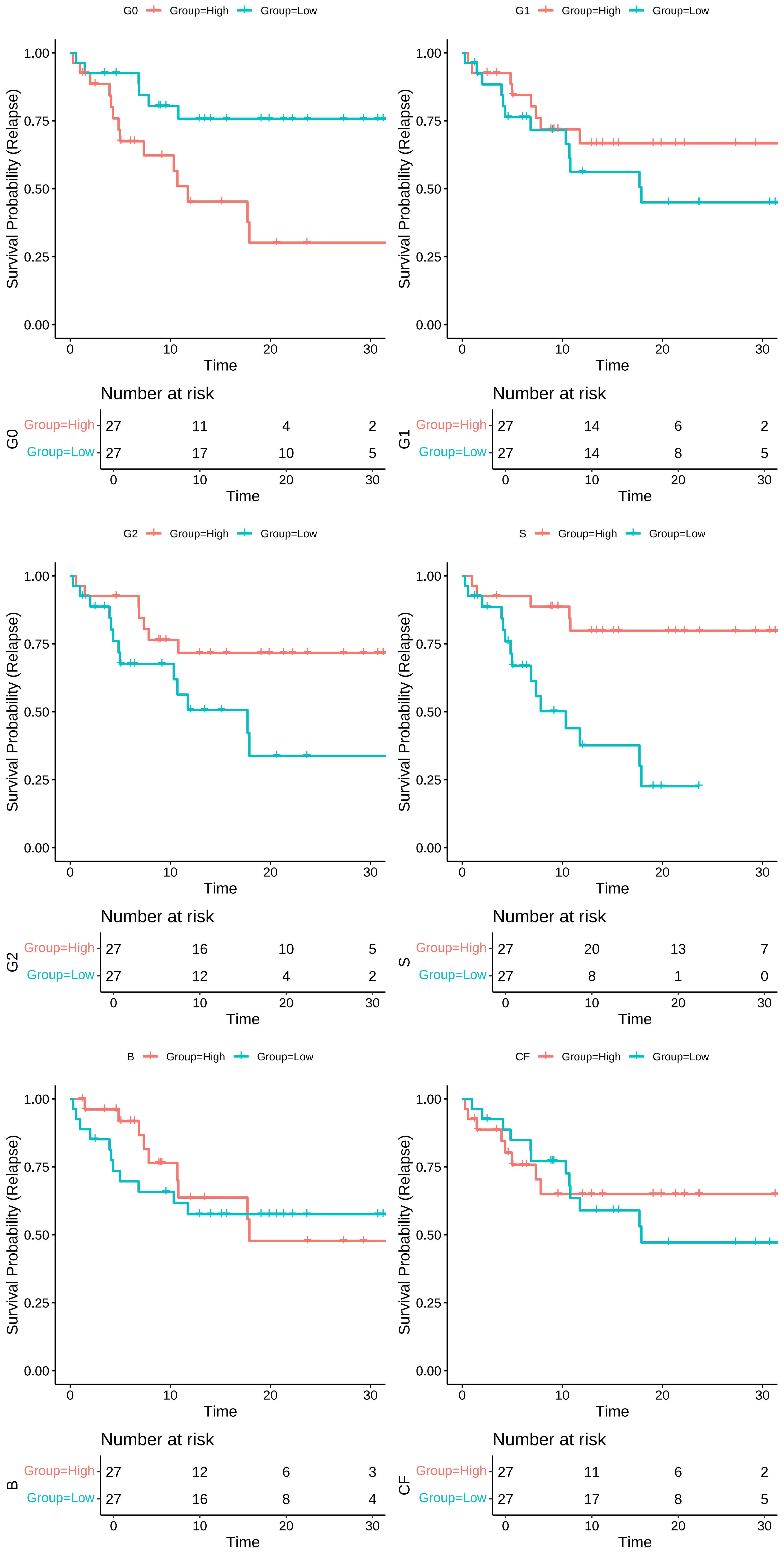

G0
Group=High
Group=Low
Survival Probability (Relapse)
1.00
0.75
0.50
0.25
0.00
0
10
20
30
Time
Number at risk
Group=High 27 11 4 2
Group=Low 27 17 10 5
G1
Group=High 27 14 6 2
Group=Low 27 14 8 5
G2
Group=High 27 16 10 5
Group=Low 27 12 4 2
S
Group=High 27 20 13 7
Group=Low 27 8 1 0
B
Group=High 27 12 6 3
Group=Low 27 16 8 4
CF
Group=High 27 11 6 2
Group=Low 27 17 8 5

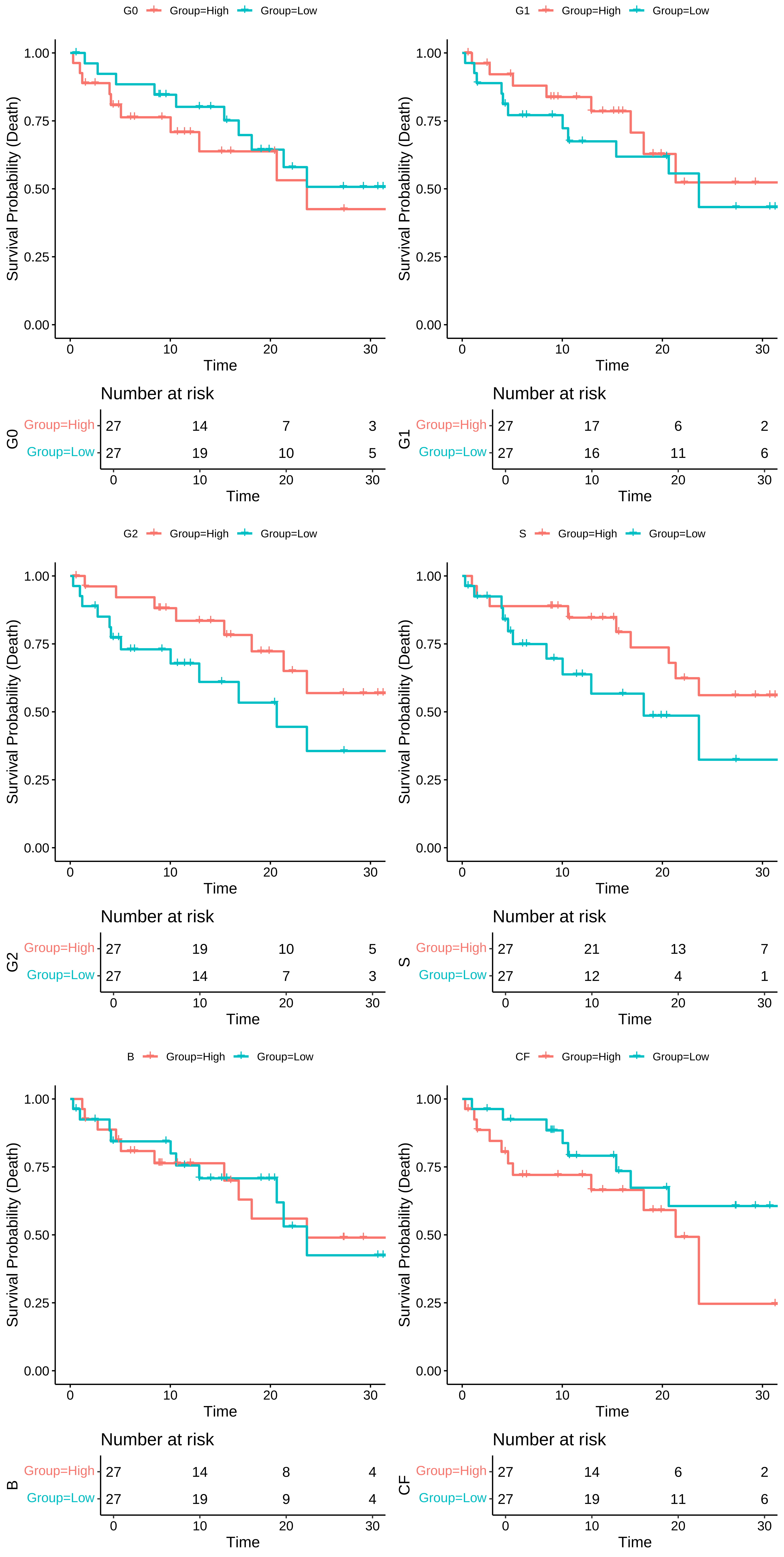
G0 Group=High Group=Low
Survival Probability (Death)
Time
Number at risk
G0
Group=High 27 14 7 3
Group=Low 27 19 10 5
G1 Group=High Group=Low
Survival Probability (Death)
Time
Number at risk
G1
Group=High 27 17 6 2
Group=Low 27 16 11 6
G2 Group=High Group=Low
Survival Probability (Death)
Time
Number at risk
G2
Group=High 27 19 10 5
Group=Low 27 14 7 3
S Group=High Group=Low
Survival Probability (Death)
Time
Number at risk
S
Group=High 27 21 13 7
Group=Low 27 12 4 1
B Group=High Group=Low
Survival Probability (Death)
Time
Number at risk
B
Group=High 27 14 8 4
Group=Low 27 19 9 4
CF Group=High Group=Low
Survival Probability (Death)
Time
Number at risk
CF
Group=High 27 14 6 2
Group=Low 27 19 11 6
